# RCVS–TCH score can predict reversible cerebral vasoconstriction syndrome in patients with thunderclap headache

**DOI:** 10.1038/s41598-021-87412-7

**Published:** 2021-04-08

**Authors:** Soohyun Cho, Mi Ji Lee, Young Eun Gil, Chin-Sang Chung

**Affiliations:** 1 Department of Neurology, Uijeongbu Eulji Medical Center, Eulji University School of Medicine, Gyeonggi, South Korea; 2 Department of Neurology, Samsung Medical Center, Sungkyunkwan University School of Medicine, Seoul, 06351 South Korea; 3 Neuroscience Center, Samsung Medical Center, Seoul, South Korea

**Keywords:** Neurology, Neurological disorders

## Abstract

Reversible cerebral vasoconstriction syndrome (RCVS) is one of the most important differential diagnosis in patients with thunderclap headache (TCH). We aimed to develop a new scoring system for RCVS in patients with TCH. We retrospectively analyzed 72 patients enrolled in the prospective study of TCH conducted in 2015–2016 (derivation set). We identified possible predictors for the diagnosis of RCVS and constructed a prediction model (RCVS–TCH score) using the multivariable logistic regression model. Diagnostic performance was validated to an independent validation set from our headache registry. The derivation set comprised 41 patients with RCVS and 31 with non-RCVS, and the validation set included 253 patients with TCH (165 with RCVS and 88 with non-RCVS). The RCVS–TCH score (range: 0–12) contained four predictors: recurrent TCHs, female sex, triggering factor for TCH (single or multi) and blood pressure surge. The C-index of RCVS–TCH score was 0.929 (95% CI = 0.874–0.984). The RCVS–TCH score ≥ 7 had a sensitivity of 80% and a specificity of 97% in discriminating RCVS from non-RCVS. In the validation set, RCVS–TCH score showed a C-index of 0.861 (95% CI = 0.815–0.908). In our study, the RCVS–TCH showed good performance, which may aid the diagnosis of RCVS among patients with TCH.

## Introduction

Reversible cerebral vasoconstriction syndrome (RCVS) is a clinical and radiological syndrome characterized by recurrent thunderclap headaches (TCHs) and reversible cerebral vasoconstriction of the cerebral arteries^1^. It is one of the most important differential diagnosis in patients with TCH because a substantial proportion of patients with RCVS can have neurological complications such as ischemic stroke, cortical subarachnoid hemorrhage (SAH), intracerebral hemorrhage, and posterior reversible encephalopathy syndrome (PRES)^2–6^. However, the diagnosis of RCVS can be challenging because of overlapping clinical features with other disorders presenting with TCH and lower sensitivity of angiography during the earlier phases of disease^7^.

Recently, the RCVS_2_ score was proposed as a diagnostic tool to distinguish RCVS in patients with intracranial vasculopathies^8^. The score includes clinical and imaging features such as recurrent/single thunderclap headache, vasoconstrictive trigger, sex, SAH, and carotid artery involvement. This score showed excellent performance in distinguishing between RCVS and intracranial vasculopathies. However, the presence of thunderclap headache is the major component of the RCVS_2_ score, and this alone can lead to the diagnosis of RCVS. Therefore, the RCVS_2_ score would not be useful for the differential diagnosis of TCH, and any patients with TCH can be falsely classified as having RCVS using this score.

Therefore, in this study, we aimed to develop a new prediction model for the diagnosis of RCVS in patients with TCH. We validated the performance of our prediction model and compared it with the RCVS_2_ score in unselected patients with TCH.

## Methods

### Study setting

For the derivation set, we retrospectively included 72 patients with TCH who participated in a prospective imaging study conducted from April 2015 to July 2016 at the Samsung Medical Center, Seoul, Korea^9^. Patients who (1) clearly remembered the mode of onset, (2) reported the time from headache onset to its peak to be < 60 s, and (3) visited within 1 month after the first attack were included, whereas those with (1) aneurysmal SAH, (2) contraindications to magnetic resonance imaging (MRI) or gadolinium enhancement, and (3) clinical manifestations suggestive of infectious meningitis were excluded. Diagnoses were based on neuroimaging findings and the International Classification of Headache Disorders (ICHD)–3 beta version^10^. Definite RCVS were diagnosed when the multifocal vasoconstrictions were not explained by SAH and normalized within 3–6 months without immunotherapy. The diagnoses of probable RCVS and primary TCH were based on ICHD–3 beta version criteria^10^. Forty-one patients had RCVS, and 31 had primary TCH or other secondary causes. The Institutional Review Board of Samsung Medical Center approved this study. All methods were performed in accordance with the relevant guidelines and regulations. Written informed consent was obtained for all patients at the inclusion visit.

### Clinical and imaging evaluation

Our protocol for evaluating TCH was described previously^9^. To summarize, the protocol was depended on the site of recruitment: emergency room (ER), outpatient headache clinic, or inpatient consultation. All patients presenting to ER underwent emergent non-contrast brain computed tomography (CT) and post-contrast CT angiography (CTA) to exclude aneurysmal SAH. In equivocal cases, lumbar puncture was done for detecting xanthochromia. In case of suspected SAH, transfemoral cerebral angiography was performed to confirm the presence of a ruptured aneurysm. The same protocol was applied in case of inpatient consultation. In the outpatient headache clinic, patients were primarily evaluated using brain MRI and MRA, whereas patients with persistent headaches were referred to the ER and the emergency protocol was then applied. For patients who were suspected of having RCVS, neuroimaging was followed-up for 3–6 months to confirm reversibility of vasoconstrictions.

We extracted clinical data of patients from a structured questionnaire, which included headache characteristics specifically designed for the evaluation of TCHs^11^. We collected information on the recurrence pattern (single or recurrent TCHs), triggering factors (situations or activities known to trigger TCH in RCVS, such as sexual activity, exertion, Valsalva maneuvers, emotion, bending, bathing and/or showering), the presence of a premorbid migraine, and blood pressure (BP) surge defined as systolic BP of > 160 mmHg during headache attacks or > 30 mmHg from baseline^3^. In the case of RCVS, the cause was classified into idiopathic or secondary (having medical conditions known to cause RCVS, such as postpartum period or vasoactive drugs). We investigated neurological manifestations such as transient focal neurological symptoms and seizure reported by patients or reliable informants. We assessed brain MRI and MRA to analyze neurological complications such as ischemic stroke, hemorrhage (parenchymal or subarachnoid) and PRES, and the distribution of vasoconstriction. We also analyzed other structural or vascular lesions that could be the secondary cause of TCH occurrence.

### Development of a new prediction model (the RCVS–TCH score)

To develop a prediction model for RCVS using the derivation set, we performed the univariable logistic regression analysis of clinical and imaging factors associated with RCVS. Then, we selected a list of candidate predictors of RCVS that were significant in univariable logistic regression with a *p* value of < 0.05. Selected variables were entered into the multivariable logistic regression model to assign a score value to each variable. The integer value closest to the beta coefficient of each variable was assigned as the weighted score. The sum of the weighted score was named as the “RCVS–TCH score.” For the diagnostic performance of the RCVS–TCH score, discriminative power was assessed using the concordance index (C-index), and calibration power was assessed by using the Hosmer and Lemeshow goodness-of-fit test^12^. The specificity, sensitivity, and positive and negative predictive values were calculated by analyzing the receiver operating characteristic curve (ROC). The Youden index was used to determine the optimal cut-off score. The accuracy and likelihood ratio were calculated for a cut‐off score based on the sensitivity and specificity^13^.

### Validation of the RCVS–TCH score and comparison with the RCVS_2_ score

For validation, we screened patients from the prospective headache registry of the Samsung Medical Center and extracted 253 patients with TCH who completed neuroimaging work-up within 1 month after onset. Of them, 165 had RCVS, 72 had primary TCH, and 16 had other secondary causes of TCH including intracranial artery dissection (n = 9), sentinel headache (n = 5), cardiac cephalalgia (n = 1) and airplane headache with sinus barotrauma (n = 1). These patients served as an independent validation set. Using this validation set, the diagnostic performance of the RCVS–TCH score was validated and compared with that of the RCVS_2_ score^8^.

As mentioned earlier, the RCVS_2_ score comprises five predictors. The RCVS_2_ score is the sum of the component scores (range − 2 to + 10), and a higher RCVS_2_ score indicates a higher possibility of RCVS. A total RCVS_2_ score of ≥ 5 was suggested as a cut-off score for diagnosing RCVS. Because “vasoconstrictive trigger,” one of the RCVS_2_ score components, might be confused with “triggering factor” for TCH, we used “etiology of RCVS” instead of vasoconstrictive trigger.

### Statistical analysis

Data are presented as number (percentage) or median (interquartile range, IQR), unless otherwise specified. Categorical variables were compared using the Chi-square test or Fisher’s exact test, and continuous variables were analyzed using the Student’s t-test or Mann–Whitney U test. Missing data were not imputed and were excluded from the associated analysis. Statistical analyses were performed using IBM SPSS 22.0 (IBM, Inc.) and R 3.6.0 (Vienna, Austria; http://www.R-project.org/). A two-tailed *p *value of < 0.05 was considered significant.

## Results

### Patients

Demographics and clinical characteristics of patients in the derivation and validation sets are shown in Table 1. When the RCVS group was compared with the non-RCVS group, female predominance was observed. Recurrent TCHs were reported in 181 (87.9%) patients. Triggering factors for TCH were reported in 163 (79.1%) patients. Among them, 82 (38.9%) patients reported two or more triggering factors. BP surge was observed in 82 (39.8%) patients. When compared with the non-RCVS, female sex, recurrent TCHs, multi-triggers for TCH, and BP surge were more frequently seen in the RCVS group in both datasets. Nine (4.4%) patients with RCVS had secondary causes of RCVS.

**Table 1:** Demographics and characteristics of all eligible patients.

	Derivation set (n = 72)			Validation set (n = 253)		
	RCVS (n = 41)	Non-RCVS (n = 31)	*p*	RCVS (n = 165)	Non-RCVS (n = 88)	*p*
Age	51 (45–59)	43 (35–56)	0.069	51 (44–56)	53 (44–59)	0.183
Sex (female)	36 (87.8)	22 (71)	0.014	128 (77.6)	49 (55.7)	< 0.001
Recurrent TCHs	33 (80.5)	9 (29)	< 0.001	148 (89.7)	50 (56.8)	< 0.001
**Diagnosis of RCVS**						
Definite RCVS	29 (70.7)			128 (77.6)		
Probable RCVS	12 (29.3)			37 (28.9)		
**Etiology of RCVS**						
Idiopathic	35 (85.4)			162 (98.2)		
Postpartum	3 (7.3)			0 (0.0)		
Vasoactive drugs	3 (7.3)			3 (1.8)		
**Triggering factors for TCH**						
Single	13 (31.7)	4 (12.9)	0.131	68 (41.2)	30 (34.1)	0.268
Multiple	21 (51.2)	4 (12.9)	0.001	61 (37.0)	10 (11.4)	< 0.001
Premorbid migraine	4 (9.8)	5 (16.1)	0.485	29 (17.6)	12 (13.6)	0.418
Focal neurological deficit	3 (7.3)	4 (12.9)	0.454	17 (10.3)	7 (8.0)	0.544
Seizure	2 (4.9)	0 (0.0)	0.503	1 (0.6)	0 (0.0)	> 0.999
Ischemic stroke	3 (7.3)	0 (0.0)	0.202	3/158 (1.8)	1/78 (1.1)	> 0.999
Parenchymal haemorrhage	0 (0.0)	0 (0.0)		1 (0.6)	0 (0.0)	> 0.999
PRES	4 (9.8)	0 (0.0)	0.129	1 (0.6)	0 (0.0)	> 0.999
SAH	2 (4.9)	0 (0.0)	0.503	8 (4.8)	3 (3.4)	0.756
Convexity SAH	2 (4.9)	0 (0.0)		8 (4.8)	2 (2.3)	
Non-convexity SAH	0 (0.0)	0 (0.0)		0 (0.0)	1 (1.1)	
Carotid artery involvement	2 (4.9)	0 (0.0)	0.503	10 (6.1)	7 (8.0)	0.478
BP surge	19 (46.3)	1 (3.2)	< 0.001	72/154 (46.8)	7/77 (9.1)	< 0.001

Data are presented as median (IQR) or number (percent).

*BP* blood pressure, *ICH* intracerebral hemorrhage, *PRES* posterior reversible encephalopathy syndrome, *SAH* subarachnoid hemorrhage, *TCH* thunderclap headache.

### Development of a prediction model: the RCVS–TCH score

In the univariable logistic regression analysis of the derivation set, recurrent TCHs (Beta = 2.31, OR = 10.08, 95% CI = 3.37–30.13, *p* < 0.001), female sex (Beta = 1.65, OR = 5.18, 95% CI = 1.27–21.19, *p* = 0.022), the presence of triggering factors for TCH (single, Beta = 2.37, OR = 10.68, 95% CI = 2.62–43.48, *p* = 0.001; multiple, Beta = 2.85, OR = 17.25, 95% CI = 4.41–67.44, *p* = 0.001), and BP surge (Beta = 3.25, OR = 25.91, 95% CI = 3.22–208.38, *p* = 0.002) were associated with RCVS. Age, etiology of RCVS, carotid artery involvement, and neurological complications were not significant.

**Figure 1:**
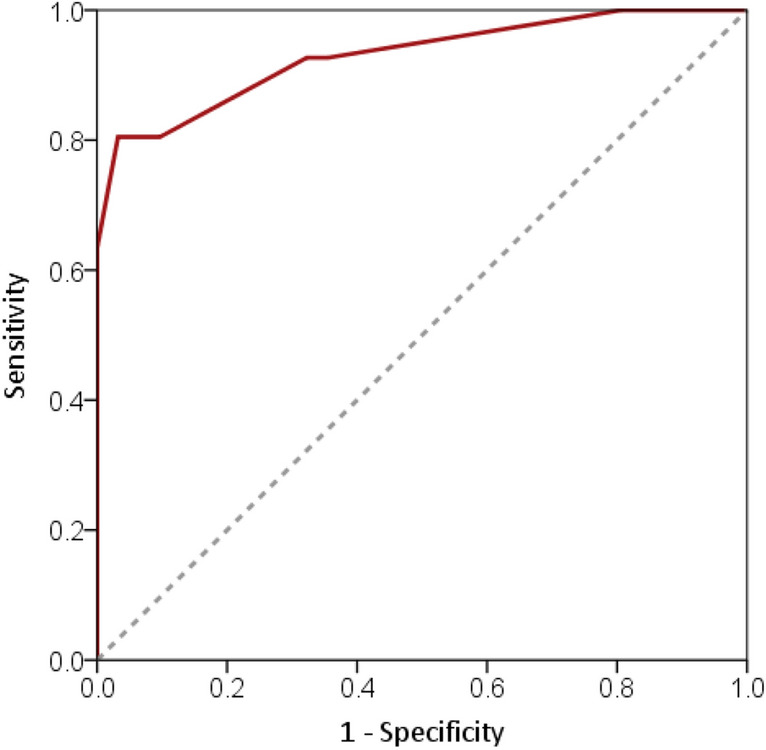
Receiver operating characteristic curve analysis for the diagnosis of reversible cerebral vasoconstriction syndrome (RCVS). Data were analyzed using the derivation data set. Solid red curve represents the RCVS–TCH score with Area Under the Curve (AUC) of 0.929 (95% CI = 0.874–0.984). Diagonal dashed line is a reference line (AUC = 0.5).

Table 2 shows results of multivariable regression analysis and score of the developed prediction model for the diagnosis of RCVS: RCVS–TCH score (range: 0–12). The ROC curve of RCVS–TCH score in the derivation data set was shown in Fig. 1. The C-index was 0.929 (95% CI = 0.874–0.984), and the Hosmer and Lemeshow goodness of fit was not significant (χ^2^ = 3.651, *p* = 0.887). RCVS–TCH score ≥ 7 was selected as the cut-off score for the diagnosis of RCVS. RCVS–TCH score ≥ 7 yielded an accuracy of 87%, a positive likelihood ratio of 26.7, a negative likelihood ratio of 0.2, a sensitivity of 80%, a specificity of 97%, and a positive predictive value of 97% and negative predictive value of 79% (Table 3).

**Table 2:** Multivariable logistic regression analysis of clinical factors associated with diagnosis of RCVS and RCVS–TCH score.

	Beta	Odds ratio	95% CI	*p*	Score
**Pattern of TCHs**					
Recurrent	1.82	6.15	1.25–30.27	0.026	2
Single	Ref				0
**Sex**					
Female	2.73	15.31	1.80–130.07	0.012	3
Male	Ref				0
**Triggering factor for TCH**					
Multiple	2.82	16.84	2.29–123.92	0.006	3
Single	2.32	10.23	1.47–71.02	0.019	2
None	Ref				0
**BP surge**					
Present	4.15	63.39	2.82–1427.0	0.009	4
Absent	Ref				0
				Range	0–12

**Table 3:** RCVS–TCH score performance.

Total scores	Derivation set				Validation set			
	Sensitivity (%)	Specificity (%)	PPV (%)	NPV (%)	Sensitivity (%)	Specificity (%)	PPV (%)	NPV (%)
9	41	100	100	56	35	100	100	44
8	63	100	100	67	60	91	93	53
7	80	97	97	79	77	78	88	61
6	80	90	92	78	81	75	87	63
5	93	68	79	88	90	52	79	66
4	93	65	78	87	97	39	76	73

*PPV* positive predictive value, *NPV* negative predictive value.

### Diagnostic performance of the RCVS–TCH and RCVS_2_ scores

**Figure 2:**
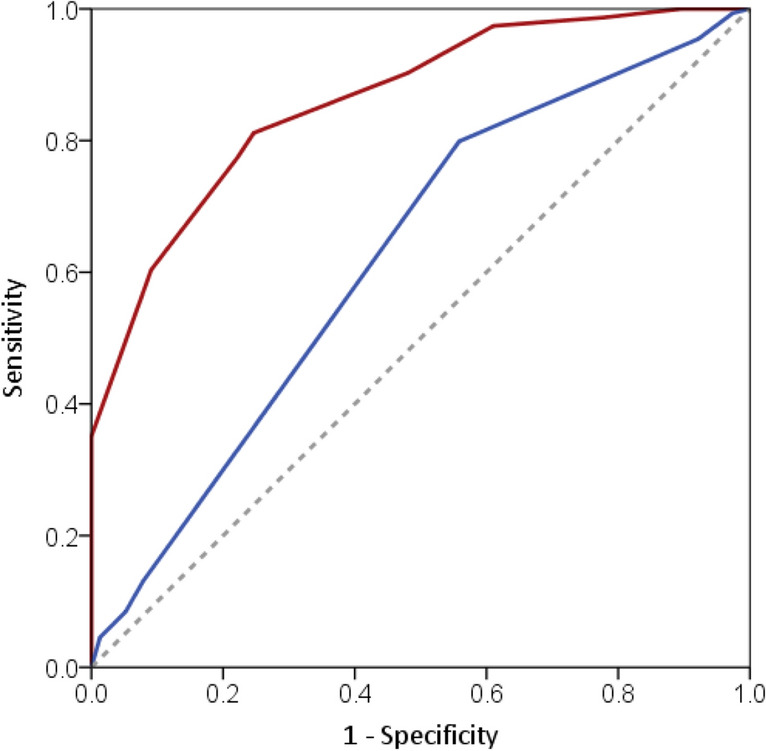
Receiver operating characteristic curve (ROC) analysis for the diagnosis of reversible cerebral vasoconstriction syndrome (RCVS). Data were analyzed using the validation data set. Solid red line represents the ROC of RCVS–TCH score with Area Under the Curve (AUC) of 0.861 (95% CI = 0.815–0.908). Solid blue line represents the ROC of RCVS_2_ score with AUC of 0.601 (95% CI = 0.526–0.677). Diagonal dashed line us a reference line (AUC = 0.5).

**Figure 3:**
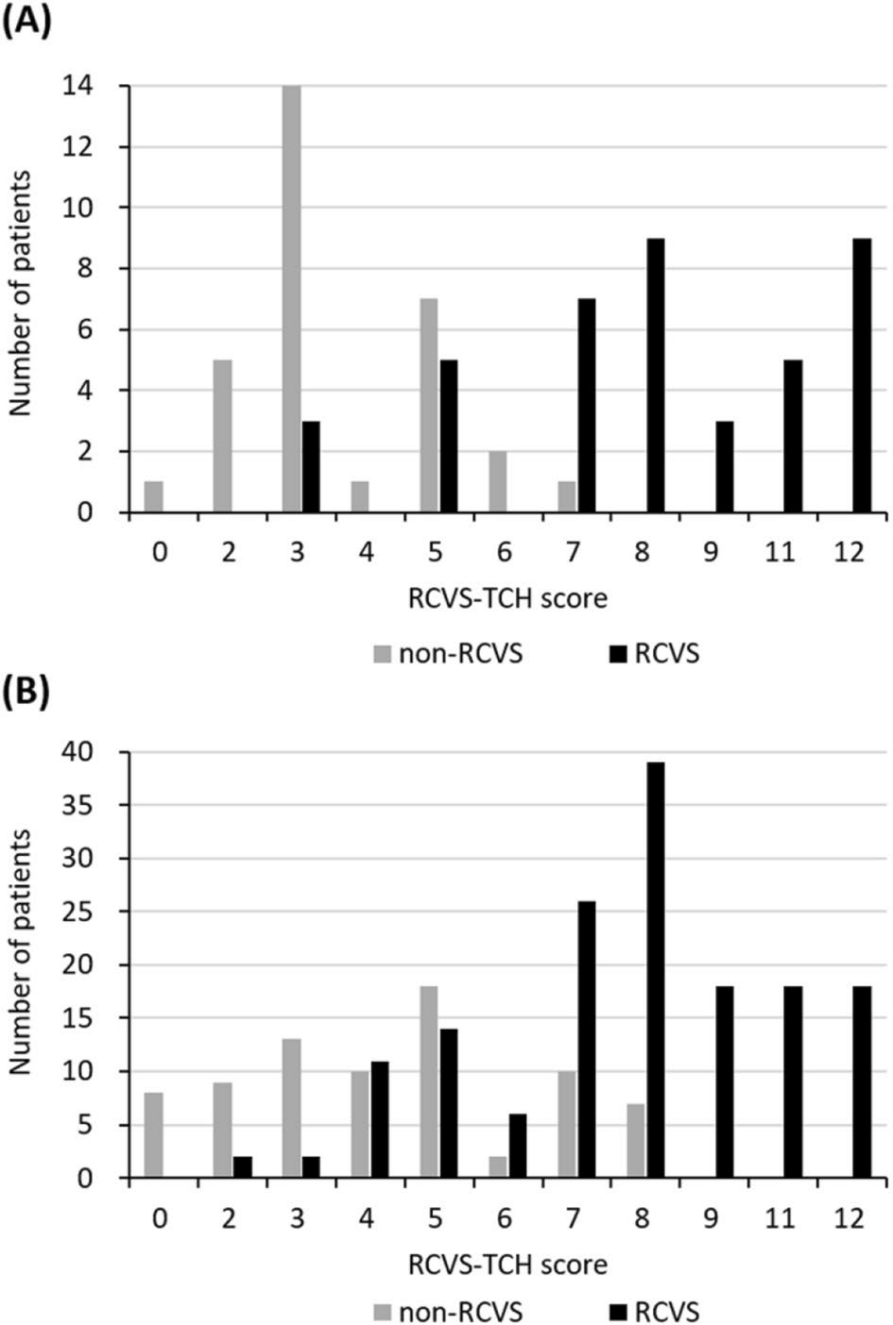
Distribution of the RCVS–TCH scores in the derivation and validation datasets. Histograms show the distribution of the RCVS–TCH score between the RCVS and non-RCVS groups in each derivation (**A**) and validation (**B**) set. Black bars indicate the number of patients with RCVS, and grey bars indicate the number of patients with non-RCVS.

The RCVS–TCH score was validated using the validation data set. The C-index was 0.861 (95% CI = 0.815–0.908), and the Hosmer and Lemeshow goodness of fit was not significant (χ^2^ = 7.038, *p* = 0.533). RCVS–TCH score ≥ 7 yielded an accuracy of 77%, a positive likelihood ratio of 3.5, a negative likelihood ratio of 0.3, a sensitivity of 77%, a specificity of 78%, and a positive predictive value of 88% and negative predictive value of 61%. When the RCVS_2_ score was validated using the same dataset, the C-index was 0.601 (95% CI = 0.526–0.677). RCVS_2_ score ≥ 5 yielded an accuracy of 65%, a positive likelihood ratio of 1.0, a negative likelihood ratio of 0.5, a sensitivity of 96%, a specificity of 8%, and a positive predictive value of 66% and negative predictive value of 50%. The ROC curves between RCVS–TCH score and RCVS_2_ score were compared in Fig. 2. Figure 3 shows the distribution of scores in the RCVS and non-RCVS groups. In the derivation set, 33 patients with RCVS and only one non-RCVS patient (score 7, the patient with primary TCH) (Fig. 3A) scored ≥ 7. In the validation data set, 119 patients with RCVS, 12 with primary TCH, and 5 with other secondary causes of TCH (sentinel headache, n = 2 and intracranial arterial dissection without RCVS, n = 3) scored ≥ 7 (Fig. 3B).

## Discussions

We developed a new prediction model, the RCVS–TCH score, to aid the diagnosis of RCVS in patients with TCH. Our model reliably predicted the diagnosis of RCVS based on clinical features. The RCVS–TCH score showed a superior performance to the RCVS_2_ score.

In this study, the RCVS–TCH score showed a good performance, with a high discriminative power and good calibration power. Our score also showed good accuracy and strong likelihood ratio with high sensitivity and specificity to predict RCVS among patients with TCH. In contrast, the RCVS_2_ score showed poor performance with poor accuracy in this patient population. In a previous study, the RCVS_2_ score showed an excellent performance to predict RCVS in patients with arteriopathies^8^. However, the score of TCH was highest, which led that the presence of TCH alone would be enough to classify RCVS under the RCVS_2_ score system. However, several disorders other than RCVS can cause TCH, but the RCVS_2_ score would not be useful in the differential diagnosis of TCH. Furthermore, aneurysmal SAH is most important differential diagnosis because it requires an emergent therapeutic approach, otherwise it would cause significant morbidity or even death^14,15^. This should be considered first in patients with TCH. Brain non-contrast CT, CTA, and lumbar puncture have high specificities and sensitivities to exclude aneurysmal SAH, so they should be conducted before MRI and MRA when aneurysmal SAH is suspected^16^. This cannot be replaced by clinical scoring such as the RCVS_2_ and RCVS–TCH scores. We recruited patients with TCH after carefully excluding aneurysmal SAH as a validation set. Among them, 33% had disorders other than RCVS. This finding again highlights that not all TCHs are RCVS. The involvement of the internal carotid artery was not included in our score model. This might be because our study participants were all Asians in whom asymptomatic intracranial atherosclerosis is common^17^. Nevertheless, our finding is in line with that of Rocha et al.’s study^8^ because the internal carotid artery was rarely involved in RCVS, even though it was similarly rare in patients with non-RCVS in our study.

In this study, female sex, recurrent TCHs, and triggering factors for TCH were used for the development of the RCVS–TCH score. This is in line with previous studies reporting that 81–90% of patients with RCVS were women, 78–100% had recurrent TCHs, and 75–80% had one or more triggering factors for TCH^2,6,18^. Among these clinical factors, recurrent TCHs and the presence of triggers are included in the ICHD-3 criteria for acute headache attributed to RCVS (6.7.3.1) or probable RCVS (6.7.3.2)^10,19^. While our study results support the feasibility of the ICHD-3 criteria, the addition of female sex could be considered in the next version of ICHD^10,19^. Although we reported the importance of female sex for the diagnosis of RCVS, it should not be overlooked that despite a small proportion, male sex could be diagnosed with RCVS. We also found that multiple triggering factors were more associated with RCVS compared to single triggering factor. This finding suggests that the multiplicity of triggering factors might be worthy to be considered when revising the diagnostic criteria of RCVS.

In our study, BP surge was observed in 47% of patients with RCVS, which is similar to the findings in previous studies^2,6^. BP surge was the most powerful predictor of RCVS in the RCVS–TCH score. The pathophysiology of BP surge in RCVS has not been fully elucidated, but it is thought that BP surge might result from either sympathetic overactivity or a stress response to excruciating headaches^20^. In our study, only a small proportion of non-RCVS patients with TCH had BP surge, suggesting that BP surge may be caused not by a response to severe headache but by the unique pathophysiology of RCVS.

Timely and accurate diagnosis of RCVS is necessary to ensure appropriate patient care and avoid unnecessary diagnostic tests. Early recognition of RCVS is important because RCVS can cause neurological complications such as intracranial hemorrhage and PRES during the early stage^6,21^. The diagnosis of RCVS is based on typical clinical features and reversibility of multiple vasoconstrictions. However, the diagnosis is often challenging because RCVS can have diverse clinical manifestations and angiographic findings can be negative during the early phase of disease^22,23^. A previous study showed that the first angiogram within 1 week was normal in up to 30% of patients who were suspicious of RCVS^6^. Furthermore, the diagnosis of RCVS can be challenging because its clinical features overlap with other thunderclap headache disorders^24–27^. Even though clinical and angiographic features are compatible with RCVS, the definitive diagnosis of RCVS can be delayed until reversibility of vasoconstriction is confirmed. It can be difficult to differentiate vasoconstrictions of RCVS with those of other various diseases such as intracranial atherosclerosis, intracranial dissection, primary angiitis of the central nervous system, and moyamoya disease.

In our study, the RCVS–TCH score showed high specificity and sensitivity for discriminating RCVS in patients with TCH. We suggest using the RCVS–TCH score for the differential diagnosis of TCH after aneurysmal SAH has been excluded using appropriate diagnostic methods. The RCVS–TCH score can be useful particularly when the angiographic finding is negative or equivocal. The RCVS_2_ score was not discriminative of RCVS in patients with TCH. On the other hand, RCVS_2_ score would be helpful in the differential diagnosis of intracranial angiopathies^8^. These scores may help physicians to determine the necessity of further work-ups such as repeated noninvasive angiography, transfemoral conventional angiography, and brain biopsy and initial tentative treatment (calcium-channel blockers for RCVS vs. steroid for angiitis).

This is the first study to develop a prediction model for the diagnosis of RCVS in patients with TCH. To develop this model, we used high-quality data collected in a previous prospective study. Validation was performed using a large group of unselected patients with TCHs. Nevertheless, there are several limitations to this study. First, the sample size was relatively small in the derivation set. In addition, the data were obtained from a single center and a single ethnicity (i.e. Asians). These factors may limit the generalizability of study findings. However, we validated using large, unselected and prospectively recruited patients and the prevalence of key factors used in the RCVS–TCH score was similar between our cohort and others. Nevertheless, future studies using this scoring system on a more heterogeneous group of patients are needed. Second, patients were included after excluding aneurysmal subarachnoid hemorrhages. So, our study findings are not applicable to all patients with TCH presenting to the emergency department. Careful exclusion of aneurysmal SAH should be performed first. Third, non-RCVS patients mainly comprised those with primary TCH. Considering the low prevalence of other secondary causes among patients with TCH, it might be that there were few patients with secondary causes. External validation is required to confirm that this model will be useful to distinguish RCVS from other secondary causes. Fourth, it was challenging to distinguish RCVS from intracranial artery dissection because 25% of patients with intracranial artery dissection were more than cut-off score. This may be because of similar clinical features between intracranial artery dissection and RCVS. In addition to our results, future studies should investigate the high-resolution vessel wall imaging to discriminate between them more accurately. Lastly, in terms of patient population, our study and the study of Rocca et al. differ that our patient population was those with thunderclap headache whereas RCVS_2_ score was proposed as a diagnostic tool to distinguish RCVS in patients with intracranial vasculopathies (regardless of the presence of headache). These important differences motivated the design and conceptualization of our study. We recommend to choose the RCVS–TCH score or RCVS_2_ score according to the clinical setting. The RCVS–TCH score should be considered first in the differential diagnosis of thunderclap headache, whereas RCVS_2_ score would be helpful in the differential diagnosis of intracranial angiopathies.

## Conclusions

The RCVS–TCH score, a new prediction model for RCVS among patients with TCH, showed good performance in distinguishing RCVS from primary TCH or other secondary causes of TCH. Our findings would aid the diagnosis of RCVS among patients with TCH when the aneurysmal subarachnoid hemorrhages were excluded.

## Data Availability

Any data not published within this article will be shared, in an anonymized data, will be shared by request from any qualified investigator.
